# *In Vitro* Activity of Gepotidacin against Gram-Negative and Gram-Positive Anaerobes

**DOI:** 10.1128/aac.02165-21

**Published:** 2022-02-15

**Authors:** Meredith A. Hackel, James A. Karlowsky, Michele A. Canino, Daniel F. Sahm, Nicole E. Scangarella-Oman

**Affiliations:** a IHMA, Schaumburg, Illinois, USA; b Department of Medical Microbiology and Infectious Diseases, Max Rady College of Medicine, University of Manitoba, Winnipeg, Manitoba, Canada; c GlaxoSmithKline, Collegeville, Pennsylvania, USA

**Keywords:** gepotidacin, triazaacenaphthylene, topoisomerase, inhibitor, anaerobes

## Abstract

Gepotidacin (formerly GSK2140944) is a first-in-class triazaacenaphthylene antibacterial currently in phase III clinical trials. When tested against Gram-negative (*n *= 333) and Gram-positive (*n *= 225) anaerobes by agar dilution, gepotidacin inhibited 90% of isolates at concentrations of 4 and 2 μg/mL, respectively. Given gepotidacin’s *in vitro* activity against the anaerobic isolates tested, further study is warranted to better understand the utility of gepotidacin in the treatment of infections caused by clinically relevant anaerobic organisms.

## INTRODUCTION

Anaerobic bacteria are etiologic agents in a wide variety of human infections and are most commonly identified as components of mixed aerobic-anaerobic infections (1). Commonly isolated anaerobes from clinical specimens include *Bacteroides* spp., *Fusobacterium* spp., *Prevotella* spp., *Porphyromonas* spp., *Actinomyces* spp., *Clostridium* spp., and Gram-positive cocci (1). Increases in resistance to commonly prescribed anti-anaerobic agents have been widely reported for clinical isolates of both Gram-negative and Gram-positive anaerobes (2–7). New antimicrobial agents with spectra of activity that target or include anaerobes via novel mechanisms of action would enhance our therapeutic armamentarium.

Gepotidacin (formerly GSK2140944) is a first-in-class triazaacenaphthylene bacterial type II topoisomerase inhibitor that is currently in phase III clinical trials as an oral treatment for uncomplicated urogenital gonorrhea (ClinicalTrials registration number NCT04010539) and uncomplicated urinary tract infections (ClinicalTrials registration number NCT04020341). Phase II clinical trials have demonstrated the efficacy of gepotidacin in the treatment of acute bacterial skin and skin structure infections (ClinicalTrials registration number NCT02045797), uncomplicated urogenital gonorrhea (ClinicalTrials registration number NCT02294682), and uncomplicated urinary tract infections (ClinicalTrials registration number NCT03568942) (8–11). Bacteria typically possess two distinct type II topoisomerases, namely, DNA gyrase and topoisomerase IV (12). DNA gyrase primarily introduces negative supercoils into DNA, mediated by the C-terminal domain of its DNA binding subunit (GyrA), while topoisomerase IV decatenates DNA and relaxes positive supercoils (12). Gepotidacin selectively inhibits both bacterial DNA gyrase and topoisomerase IV by interacting with the bacterial subunits GyrA (DNA gyrase) and ParC (topoisomerase IV) using a novel mode of binding (13–15). Specifically, molecular dynamics simulations have shown that D82 in the GyrA subunit of DNA gyrase and the homologous position D79 in the ParC subunit of topoisomerase IV form an intermolecular salt bridge with gepotidacin (16). Once bound, gepotidacin associates with uncleaved or single-stranded cleaved DNA complexes to inhibit bacterial DNA replication and cell division. Gepotidacin is active *in vitro* against fastidious and nonfastidious aerobic, Gram-positive and Gram-negative bacteria, including isolates that are resistant to fluoroquinolones and antimicrobial agents of other antimicrobial classes (8, 11, 17–19). To date, only a single study has been published on the *in vitro* activity of gepotidacin against anaerobes, and it included only Clostridium perfringens (*n *= 101; MIC_90_, 0.5 μg/mL) (18).

In the current study, 649 clinically significant anaerobic pathogens that had been previously collected by IHMA surveillance/clinical studies in North America (*n *= 315 [48.5% of isolates]) and Europe (*n = *334 [51.5% of isolates]) in 2000 to 2017 were included for testing. The anatomical sites of organism isolation included intraabdominal (*n =* 237 [36.5% of total]), skin and skin structure (*n =* 214 [33.0%]), other or unknown (*n =* 110 [16.9%]), blood (39 [6.0%]), genital (*n =* 23 [3.5%]), respiratory tract (*n =* 18 [2.8%]), and urinary tract (*n =* 8 [1.2%]). The isolate collection analyzed included Gram-negative (*n = *333) and Gram-positive (*n = *225) anaerobes. A collection of *Lactobacillus* strains (*n = *91) was also analyzed. All isolates used in this study were preserved at −70°C; the majority were collected in 2013 to 2016 (*n *= 432 [66.6% of isolates]). Isolates were identified to the species level using matrix-assisted laser desorption ionization–time of flight (MALDI-TOF) mass spectrometry (Bruker Daltronics, Bremen, Germany) (library version MBT Compass 4.1.60.).

MICs were determined by agar dilution for all isolates and by both agar dilution and broth microdilution for *Bacteroides* strains (20, 21). The 316 Gram-positive isolates tested included 91 isolates of *Lactobacillus* spp. The CLSI document M45-A3 recommends determining MICs for *Lactobacillus* spp. using cation-adjusted Mueller-Hinton broth supplemented with 2.5% to 5% laked horse blood, with panels incubated at 35°C in 5% CO_2_ for 24 to 48 h (22). However, the *Lactobacillus* isolates included in this study grew poorly in 5% CO_2_ and required anaerobic conditions for optimal growth; therefore, they were tested by anaerobic agar dilution (12), as suggested in the CLSI document M45-A3 (22). MICs generated against Gram-negative and Gram-positive isolates other than *Lactobacillus* spp. were interpreted using CLSI M100 MIC breakpoints (21). *Lactobacillus* data were reported separately from data for other Gram-positive isolates, with MICs interpreted using CLSI M45-A3 breakpoints (22).

Ceftriaxone, metronidazole, and piperacillin powders were obtained from Sigma-Aldrich (St. Louis, MO). Clindamycin, imipenem, moxifloxacin, and tazobactam powders were obtained from the U.S. Pharmacopeia (Rockville, MD). Gepotidacin was provided by GlaxoSmithKline (Collegeville, PA). All antimicrobial agents were dissolved and diluted following CLSI guidelines (21). Quality control testing was performed on each day of testing as specified by the CLSI, using Bacteroides fragilis ATCC 25825, Bacteroides thetaiotaomicron ATCC 29741, Eggerthella lenta ATCC 43055, and Clostridioides difficile ATCC 700057 (21). It is important to note that CLSI quality control MIC ranges have not yet been established for gepotidacin tested against anaerobes.

The *in vitro* activities of gepotidacin and comparator agents against Gram-negative anaerobes are shown in Table 1. The gepotidacin MIC_90_ for all Gram-negative isolates (tested by agar dilution) was 4 μg/mL. Gepotidacin was more potent on a weight basis than ceftriaxone (MIC_90_, 512 μg/mL), clindamycin (MIC_90_, >8 μg/mL), moxifloxacin (MIC_90_, 8 μg/mL), and piperacillin-tazobactam (MIC_90_, 16 μg/mL) but was less potent than imipenem (MIC_90_, 0.5 μg/mL) and metronidazole (MIC_90_, 2 μg/mL) against this collection of Gram-negative anaerobes. Gepotidacin MIC_90_ values ranged from 0.12 μg/mL for *Veillonella* spp. to 4 μg/mL for both *Bacteroides* spp. and *Prevotella* spp. The MIC_90_ for gepotidacin against the B. fragilis group was 1 doubling dilution lower when tested by broth microdilution (2 μg/mL) than when tested by agar dilution (4 μg/mL). Gepotidacin at concentrations of 1, 2, 4, 8, and 16 μg/mL inhibited 79.0%, 88.9%, 96.4%, 98.2%, and 99.1% of Gram-negative isolates (Table 2).

**TABLE 1:** *In vitro* activities of gepotidacin and comparator agents against 333 isolates of Gram-negative anaerobes

Organism group and antimicrobial agent	MIC (μg/mL)^*a*^			MIC interpretation (%)		
	MIC range	MIC_50_	MIC_90_	Susceptible	Intermediate	Resistant
All Gram negative (333 isolates)						
Gepotidacin	≤0.015 to >32	0.5	4	NA^*b*^	NA	NA
Ceftriaxone	≤8 to >512	≤8	512	58.6	8.7	32.7
Clindamycin	≤0.25 to >8	0.5	>8	73.3	5.4	21.3
Imipenem	≤0.015 to >8	0.12	0.5	99.1	0.3	0.6
Metronidazole	≤0.12 to >16	0.25	2	98.5	0	1.5
Moxifloxacin	≤0.06 to >8	0.5	8	78.1	10.8	11.1
Piperacillin-tazobactam	≤0.06 to >64	0.5	16	97.6	1.5	0.9
Bacteroides fragilis group^*c*^ (191 isolates)						
Agar dilution testing						
Gepotidacin	≤0.015 to 32	1	4	NA	NA	NA
Ceftriaxone	≤8 to >512	64	>512	34.0	13.6	52.4
Clindamycin	≤0.25 to >8	1	>8	64.4	7.9	27.8
Imipenem	0.03 to >8	0.25	1	99.0	0.5	0.5
Metronidazole	≤0.12 to 2	0.25	1	100	0	0
Moxifloxacin	0.12 to >8	1	8	71.7	11.5	16.8
Piperacillin-tazobactam	≤0.06 to >64	1	8	99.0	0	1.1
Broth microdilution testing						
Gepotidacin	≤0.015 to 16	0.5	2	NA	NA	NA
Ceftriaxone	≤4 to >512	16	256	51.3	16.8	31.9
Clindamycin	≤0.03 to >16	1	>16	64.9	8.4	26.7
Imipenem	0.03 to >8	0.12	0.5	98.4	0.5	1.1
Metronidazole	≤0.12 to 8	1	2	100	0	0
Moxifloxacin	0.12 to >8	1	8	72.3	9.4	18.3
Piperacillin-tazobactam	≤0.015 to >64	1	16	99.0	0	1
Bilophila wadsworthia (26 isolates)						
Gepotidacin	0.03 to 2	0.25	0.5	NA	NA	NA
Ceftriaxone	≤8 to ≤8	≤8	≤8	100	0	0
Clindamycin	≤0.25 to ≤0.25	≤0.25	≤0.25	100	0	0
Imipenem	≤0.015 to 0.25	≤0.015	0.12	100	0	0
Metronidazole	≤0.12 to 0.25	≤0.12	≤0.12	100	0	0
Moxifloxacin	≤0.06 to 0.5	0.25	0.5	100	0	0
Piperacillin-tazobactam	≤0.06 to 4	1	4	100	0	0
*Fusobacterium* spp.^*d*^ (25 isolates)						
Gepotidacin	≤0.015 to >32	0.12	2	NA	NA	NA
Ceftriaxone	≤8 to >512	≤8	≤8	96.0	0	4.0
Clindamycin	≤0.25 to >8	≤0.25	4	88.0	4.0	8.0
Imipenem	≤0.015 to 1	0.03	0.12	100	0	0
Metronidazole	≤0.12 to 2	≤0.12	0.5	100	0	0
Moxifloxacin	≤0.06 to 4	0.25	2	96.0	4.0	0
Piperacillin-tazobactam	≤0.06 to 32	≤0.06	0.25	100	0	0
*Porphyromonas* spp.^*e*^ (26 isolates)						
Gepotidacin	≤0.015 to >32	0.06	1	NA	NA	NA
Ceftriaxone	≤8 to 128	≤8	≤8	96.2	0	3.9
Clindamycin	≤0.25 to >8	≤0.25	>8	84.6	0	15.4
Imipenem	≤0.015 to >8	0.06	0.5	96.2	0	3.9
Metronidazole	≤0.12 to >16	≤0.12	2	96.2	0	3.9
Moxifloxacin	≤0.06 to 4	0.25	2	96.2	3.9	0
Piperacillin-tazobactam	≤0.06 to 32	0.12	8	100	0	0
*Prevotella* spp.^*f*^ (30 isolates)						
Gepotidacin	0.06 to 4	0.5	4	NA	NA	NA
Ceftriaxone	≤8 to 512	≤8	128	70.0	10.0	20.0
Clindamycin	≤0.25 to >8	≤0.25	>8	76.7	0	23.3
Imipenem	≤0.015 to 0.12	0.03	0.06	100	0	0
Metronidazole	≤0.12 to 2	0.5	2	100	0	0
Moxifloxacin	0.25 to 8	1	4	73.3	20.0	6.7
Piperacillin-tazobactam	≤0.06 to 2	≤0.06	0.25	100	0	0
Sutterella wadsworthensis (10 isolates)						
Gepotidacin	0.25 to 1	0.5	1	NA	NA	NA
Ceftriaxone	≤8 to 16	≤8	16	100	0	0
Clindamycin	0.5 to 1	1	1	100	0	0
Imipenem	0.25 to 0.5	0.5	0.5	100	0	0
Metronidazole	2 to >16	4	>16	60.0	0	40.0
Moxifloxacin	0.12 to 1	0.25	0.5	100	0	0
Piperacillin-tazobactam	32 to 64	32	64	80.0	20.0	0
*Veillonella* spp.^*g*^ (25 isolates)						
Gepotidacin	≤0.015 to 0.25	0.12	0.12	NA	NA	NA
Ceftriaxone	≤8 to >512	≤8	≤8	96.0	0	4.0
Clindamycin	≤0.25 to >8	≤0.25	>8	72.0	8.0	20.0
Imipenem	0.06 to 2	0.5	2	100	0	0
Metronidazole	0.5 to 4	2	4	100	0	0
Moxifloxacin	≤0.06 to >8	1	8	64.0	24.0	12.0
Piperacillin-tazobactam	0.25 to >64	16	64	84.0	12.0	4.0

a MIC_50_ and MIC_90_ values were calculated only for genera or species for which >10 isolates were tested.

b NA, not available. CLSI M100 MIC breakpoints are not published for this antimicrobial agent.

c Bacteroides fragilis group isolates included Bacteroides caccae (*n *= 2), Bacteroides fragilis (*n *= 114), Bacteroides ovatus (*n *= 11), Bacteroides stercoris (*n *= 3), Bacteroides thetaiotaomicron (*n *= 48), Bacteroides uniformis (*n *= 4), and Bacteroides vulgatus (*n *= 9).

d *Fusobacterium* isolates included Fusobacterium necrophorum (*n *= 3), Fusobacterium nucleatum (*n *= 17), and *Fusobacterium* not identified at the species level (*n *= 5).

e *Porphyromonas* isolates included Porphyromonas asaccharolytica (*n *= 9), Porphyromonas endodontalis (*n *= 2), Porphyromonas gingivalis (*n *= 2), Porphyromonas levii (*n *= 1), Porphyromonas somerae (*n *= 5), and *Porphyromonas* not identified at the species level (*n *= 7).

f *Prevotella* isolates included Prevotella bivia (*n *= 11), Prevotella buccae (*n *= 10), Prevotella denticola (*n *= 5), Prevotella disiens (*n *= 1), and Prevotella melaninogenica (*n *= 3).

g *Veillonella* isolates included Veillonella alcalescens (*n *= 1), Veillonella parvula (*n *= 9), and *Veillonella* not identified at the species level (*n *= 15).

**TABLE 2:** Distribution of gepotidacin and comparator MICs against Gram-negative and Gram-positive anaerobic isolates

Organism group and antimicrobial agent	No. (cumulative %) of isolates with gepotidacin MIC of:												
	≤0.015 μg/mL	0.03 μg/mL	0.06 μg/mL	0.12 μg/mL	0.25 μg/mL	0.5 μg/mL	1 μg/mL	2 μg/mL	4 μg/mL	8 μg/mL	16 μg/mL	32 μg/mL	>32 μg/mL
All Gram negative^*a*^ (333 isolates)													
Gepotidacin	17 (5.1)	9 (7.8)	22 (14.4)	33 (24.3)	40 (36.3)	67 (56.5)	75 (79.0)	33 (88.9)	25 (96.4)	6 (98.2)	3 (99.1)	1 (99.4)	2 (100)
Ceftriaxone										169 (50.8)	26 (58.6)	29 (67.3)	109 (100)
Clindamycin					129 (38.7)	47 (52.9)	41 (65.2)	27 (73.3)	18 (78.7)	71 (100)			
Imipenem	39 (11.7)	27 (19.8)	62 (38.4)	67 (58.6)	70 (79.6)	37 (90.7)	22 (97.3)	5 (98.8)	1 (99.1)	3 (100)			
Metronidazole				129 (38.7)	43 (51.7)	88 (78.1)	33 (88.0)	22 (94.6)	12 (98.2)	1 (98.5)	5 (100)		
Moxifloxacin			8 (2.4)	21 (8.7)	88 (35.1)	71 (56.5)	49 (71.2)	23 (78.1)	36 (88.9)	37 (100)			
Piperacillin-tazobactam			82 (24.6)	33 (34.5)	44 (47.7)	19 (53.5)	30 (62.5)	22 (69.1)	20 (75.1)	37 (86.2)	17 (91.3)	21 (97.6)	8 (100)
All Gram positive^*b*^ (225 isolates)													
Gepotidacin	12 (5.3)	15 (12.0)	16 (19.1)	12 (24.4)	14 (30.7)	22 (40.4)	69 (71.1)	43 (90.2)	10 (94.7)	3 (96.0)	2 (96.9)	2 (97.8)	5 (100)
Ceftriaxone										85 (37.8)	19 (46.2)	53 (69.8)	68 (100)
Clindamycin					98 (43.6)	7 (46.7)	12 (52.0)	32 (66.2)	41 (84.4)	35 (100)			
Imipenem	27 (12.0)	22 (21.8)	27 (33.8)	14 (40.0)	13 (45.8)	17 (53.3)	4 (55.1)	13 (60.9)	60 (87.6)	28 (100)			
Metronidazole				70 (31.1)	93 (72.4)	41 (90.7)	12 (96.0)	5 (98.2)	1 (98.7)		3 (100)		
Moxifloxacin			6 (2.7)	29 (15.6)	20 (24.4)	12 (29.8)	69 (60.4)	17 (68.0)	17 (75.6)	55 (100)			
Piperacillin-tazobactam			36 (16.0)	24 (26.7)	23 (36.9)	6 (39.6)	5 (41.8)	2 (42.7)	10 (47.1)	85 (84.9)	15 (91.6)	19 (100)	

a Gram-negative isolates included Bacteroides caccae (*n *= 2), Bacteroides fragilis (*n *= 114), Bacteroides ovatus (*n *= 11), Bacteroides stercoris (*n *= 3), Bacteroides thetaiotaomicron (*n *= 48), Bacteroides uniformis (*n *= 4), Bacteroides vulgatus (*n *= 9), Bilophila wadsworthia (*n *= 26), Fusobacterium necrophorum (*n *= 3), Fusobacterium nucleatum (*n *= 17), *Fusobacterium* not identified at the species level (*n *= 5), Porphyromonas asaccharolytica (*n *= 9), Porphyromonas endodontalis (*n *= 2), Porphyromonas gingivalis (*n *= 2), Porphyromonas levii (*n *= 1), Porphyromonas somerae (*n *= 5), *Porphyromonas* not identified at the species level (*n *= 7), Prevotella bivia (*n *= 11), Prevotella buccae (*n *= 10), Prevotella denticola (*n *= 5), Prevotella disiens (*n *= 1), Prevotella melaninogenica (*n *= 3), Sutterella wadsworthensis (*n *= 10), Veillonella alcalescens (*n *= 1), Veillonella parvula (*n *= 9), and *Veillonella* not identified at the species level (*n *= 15).

b Gram-positive isolates included Actinomyces europaeus (*n *= 2), Actinomyces georgiae (*n *= 1), Actinomyces israelii (*n *= 1), Actinomyces meyeri (*n *= 1), Actinomyces neuii (*n *= 3), Actinomyces odontolyticus (*n *= 3), Actinomyces radingae (*n *= 3), Actinomyces turicensis (*n *= 2), *Actinomyces* not identified at the species level (*n *= 6), Bifidobacterium adolescentis (*n *= 5), Bifidobacterium breve (*n *= 3), Bifidobacterium dentium (*n *= 4), Bifidobacterium longum (*n *= 7), Bifidobacterium pseudocatenulatum (*n *= 3), *Bifidobacterium* not identified at the species level (*n *= 4), Clostridioides difficile (*n *= 100), Collinsella aerofaciens (*n* = 5), Eggerthella lenta (*n* = 21), Eubacterium limosum (*n *= 2), Eubacterium nodatum (*n *= 1), *Eubacterium* not identified at the species level (*n *= 23), and Peptostreptococcus anaerobius (*n* = 25).

The *in vitro* activities of gepotidacin and comparator agents against Gram-positive anaerobes are shown in Table 3. The MIC_90_ for gepotidacin against all Gram-positive isolates combined was 2 μg/mL. Based on MIC_90_ values, gepotidacin was more potent on a weight basis than ceftriaxone (MIC_90_, 256 μg/mL), clindamycin (MIC_90_, >8 μg/mL), imipenem (MIC_90_, 8 μg/mL), moxifloxacin (MIC_90_, >8 μg/mL), and piperacillin-tazobactam (MIC_90_, 16 μg/mL) but was less potent than metronidazole (MIC_90_, 0.5 μg/mL). MIC_90_ values for gepotidacin against Gram-positive isolates ranged from 0.03 μg/mL for Peptostreptococcus anaerobius to >32 μg/mL for *Actinomyces* spp. C. difficile isolates (*n *= 100) had a gepotidacin MIC_90_ value of 2 μg/mL, while the remaining Gram-positive species had gepotidacin MIC_90_ values of ≤8 μg/mL. The MIC_90_ value for gepotidacin against *Lactobacillus* spp. was 1 μg/mL, and all isolates were inhibited by ≤2 μg/mL (Table 4). Gepotidacin at concentrations of 1, 2, 4, 8, and 16 μg/mL inhibited 71.1%, 90.2%, 94.7%, 96.0%, and 96.9% of Gram-positive isolates (Table 2).

**TABLE 3:** *In vitro* activities of gepotidacin and comparator agents against 225 isolates of Gram-positive anaerobes

Organism group and antimicrobial agent	MIC (μg/mL)^*a*^			MIC interpretation (%)		
	MIC range	MIC_50_	MIC_90_	Susceptible	Intermediate	Resistant
All Gram positive (225 isolates)						
Gepotidacin	≤0.015 to >32	1	2	NA^*b*^	NA	NA
Ceftriaxone	≤8 to >512	32	256	46.2	23.6	30.2
Clindamycin	≤0.25 to >8	1	>8	66.2	18.2	15.6
Imipenem	≤0.015 to >8	0.5	8	87.6	11.6	0.9
Metronidazole	≤0.12 to >16	0.25	0.5	98.7	0	1.3
Moxifloxacin	≤0.06 to >8	1	>8	68.0	7.6	24.4
Piperacillin-tazobactam	≤0.06 to 32	8	16	100	0	0
*Actinomyces* spp.^*c*^ (22 isolates)						
Gepotidacin	0.06 to >32	1	>32	NA	NA	NA
Ceftriaxone	≤8 to 16	≤8	16	100	0	0
Clindamycin	≤0.25 to >8	≤0.25	>8	81.8	0	18.2
Imipenem	≤0.015 to 0.25	0.06	0.12	100	0	0
Metronidazole	≤0.12 to >16	≤0.12	≤0.12	90.9	0	9.1
Moxifloxacin	≤0.06 to 8	1	2	90.9	4.6	4.6
Piperacillin-tazobactam	≤0.06 to 1	0.12	1	100	0	0
*Bifidobacterium* spp.^*d*^ (26 isolates)						
Gepotidacin	0.03 to >32	0.25	0.5	NA	NA	NA
Ceftriaxone	≤8 to 16	≤8	16	100	0	0
Clindamycin	≤0.25 to >8	≤0.25	≤0.25	96.2	0	3.9
Imipenem	≤0.015 to 0.5	0.03	0.12	100	0	0
Metronidazole	≤0.12 to 0.5	≤0.12	0.25	100	0	0
Moxifloxacin	0.12 to >8	2	8	73.1	15.4	11.5
Piperacillin-tazobactam	≤0.06 to 0.5	≤0.06	0.25	100	0	0
Clostridioides difficile (100 isolates)						
Gepotidacin	0.12 to 8	1	2	NA	NA	NA
Ceftriaxone	16 to >512	32	128	1.0	51.0	48.0
Clindamycin	≤0.25 to >8	4	>8	37.0	40.0	23.0
Imipenem	0.12 to >8	4	8	72.0	26.0	2.0
Metronidazole	0.25 to 2	0.25	1	100	0	0
Moxifloxacin	0.25 to >8	1	>8	56.0	7.0	37.0
Piperacillin-tazobactam	0.25 to 16	8	8	100	0	0
Collinsella aerofaciens (5 isolates)						
Gepotidacin	0.06 to 0.06			NA	NA	NA
Ceftriaxone	≤8 to ≤8			100	0	0
Clindamycin	≤0.25 to ≤0.25			100	0	0
Imipenem	0.03 to 0.06			100	0	0
Metronidazole	≤0.12 to ≤0.12			100	0	0
Moxifloxacin	0.25 to >8			60.0	0	40.0
Piperacillin-tazobactam	0.12 to 1			100	0	0
Eggerthella lenta (21 isolates)						
Gepotidacin	0.06 to 32	1	4	NA	NA	NA
Ceftriaxone	32 to >512	512	>512	0	9.5	90.5
Clindamycin	≤0.25 to >8	≤0.25	>8	85.7	0	14.3
Imipenem	0.12 to 0.5	0.5	0.5	100	0	0
Metronidazole	≤0.12 to 0.5	0.5	0.5	100	0	0
Moxifloxacin	0.12 to >8	0.25	>8	66.7	4.8	28.6
Piperacillin-tazobactam	8 to 32	32	32	100	0	0
*Eubacterium* spp.^*e*^ (26 isolates)						
Gepotidacin	0.03 to 4	0.25	2	NA	NA	NA
Ceftriaxone	≤8 to 512	≤8	16	96.2	0	3.9
Clindamycin	≤0.25 to >8	≤0.25	1	92.3	3.9	3.9
Imipenem	≤0.015 to 0.5	≤0.015	0.25	100	0	0
Metronidazole	≤0.12 to >16	≤0.12	0.5	96.2	0	3.9
Moxifloxacin	≤0.06 to >8	0.25	>8	88.5	0	11.5
Piperacillin-tazobactam	≤0.06 to 32	≤0.06	1	100	0	0
Peptostreptococcus anaerobius (25 isolates)						
Gepotidacin	≤0.015 to 0.06	0.03	0.03	NA	NA	NA
Ceftriaxone	≤8 to 16	≤8	≤8	100	0	0
Clindamycin	≤0.25 to >8	≤0.25	>8	88.0	0	12.0
Imipenem	0.06 to 2	0.12	2	100	0	0
Metronidazole	0.25 to 0.5	0.5	0.5	100	0	0
Moxifloxacin	0.12 to 8	0.12	8	72.0	16.0	12.0
Piperacillin-tazobactam	0.12 to 16	0.25	16	100	0	0

a MIC_50_ and MIC_90_ values were calculated only for genera or species for which >10 isolates were tested.

b NA, not available. CLSI M100 MIC breakpoints are not published for this antimicrobial agent.

c *Actinomyces* isolates included Actinomyces europaeus (*n *= 2), Actinomyces georgiae (*n *= 1), Actinomyces israelii (*n *= 1), Actinomyces meyeri (*n *= 1), Actinomyces neuii (*n *= 3), Actinomyces odontolyticus (*n *= 3), Actinomyces radingae (*n *= 3), Actinomyces turicensis (*n *= 2), and *Actinomyces* not identified at the species level (*n *= 6).

d *Bifidobacterium* isolates included Bifidobacterium adolescentis (*n *= 5), Bifidobacterium breve (*n *= 3), Bifidobacterium dentium (*n *= 4), Bifidobacterium longum (*n *= 7), Bifidobacterium pseudocatenulatum (*n *= 3), and *Bifidobacterium* not identified at the species level (*n *= 4).

e *Eubacterium* isolates included Eubacterium limosum (*n *= 2), Eubacterium nodatum (*n *= 1), and *Eubacterium* not identified at the species level (*n *= 23).

**TABLE 4:** *In vitro* activities of gepotidacin and comparator agents against 91 *Lactobacillus* isolates tested under anaerobic conditions using agar dilution^*a*^

Antimicrobial agent	MIC (μg/mL)			MIC interpretation (%)^*b*^		
	MIC range	MIC_50_	MIC_90_	Susceptible	Intermediate	Resistant
Gepotidacin	≤0.015 to 2	0.5	1	NA^*c*^	NA	NA
Ceftriaxone	≤8 to 256	32	64	NA	NA	NA
Clindamycin	≤0.25 to >8	≤0.25	4	74.7	3.3	22.0
Imipenem	≤0.015 to 8	0.25	2	68.1	2.2	29.7
Metronidazole	≤0.12 to >16	>16	>16	NA	NA	NA
Moxifloxacin	≤0.06 to >8	0.5	4	NA	NA	NA
Piperacillin-tazobactam	≤0.06 to 8	1	4	NA	NA	NA

a *Lactobacillus* isolates included Lactobacillus acidophilus (*n *= 1), Lactobacillus crispatus (*n *= 3), Lactobacillus fermentum (*n *= 5), Lactobacillus gasseri (*n *= 21), Lactobacillus iners (*n *= 2), Lactobacillus jensenii (*n *= 6), Lactobacillus plantarum (*n *= 1), Lactobacillus rhamnosus (*n *= 19), and *Lactobacillus* not identified at the species level (*n *= 33).

b Clindamycin and imipenem MICs were interpreted using MIC breakpoints published in the CLSI document M45-A3 (22).

c NA, not available. CLSI M100 MIC breakpoints are not published for this antimicrobial agent.

Gepotidacin demonstrated potent *in vitro* activity against the majority of common, clinically relevant Gram-negative and Gram-positive anaerobes. Given the extent of gepotidacin’s *in vitro* activity against the isolates tested in this study, further analysis is warranted to fully assess the scope of activity of this novel option for the treatment of infections caused by anaerobes.
